# Advances in Donkey Disease Surveillance and Microbiome Characterization in China

**DOI:** 10.3390/microorganisms13040749

**Published:** 2025-03-26

**Authors:** Muhammad Zahoor Khan, Yan Li, Mingxia Zhu, Mengmeng Li, Tongtong Wang, Zhenwei Zhang, Wenqiang Liu, Qingshan Ma, Changfa Wang

**Affiliations:** College of Agriculture and Biology, Liaocheng University, Liaocheng 252000, China; zahoorkhan@lcu.edu.cn (M.Z.K.);

**Keywords:** donkey microbial community, equid pathogens, health biomarkers, microbial diversity, veterinary epidemiology, public health

## Abstract

This review article highlights the surveillance of bacterial, viral, and parasitic diseases in donkey populations in China. Key findings highlight significant threats from Equine herpesviruses (EHV-8 and EHV-1), which cause encephalitis, abortion, and respiratory distress. Several parasitic infections including *Giardia duodenalis*, *Cryptosporidium* spp., *Enterocytozoon bieneusi*, and *Toxoplasma gondii* present important zoonotic concerns across multiple regions of China. Additionally, this review synthesizes current knowledge on donkey microbiota across various body sites and examines their functional significance in health and disease. The complex relationship between the microbiota and host health represents a critical area of research in donkeys. Recent molecular advancements have enhanced our understanding of the diverse microbial ecosystems inhabiting different body sites in donkeys and their profound impact on health outcomes. As single-stomach herbivores, donkeys possess complex microbial communities throughout their digestive tracts that are essential for intestinal homeostasis and nutritional processing. Significant variations in microbiota composition exist across different intestinal segments, with the hindgut displaying greater richness and diversity compared to the foregut. Beyond the digestive system, distinct microbial profiles have been characterized across various body sites including the skin, oral cavity, reproductive tract, and body secretions such as milk. The health implications of donkey microbiota extend to critical areas including nutrition, immune function, and disease susceptibility. Research demonstrates how dietary interventions, environmental stressors, and physiological states significantly alter microbial communities, correlating with changes in inflammatory markers, antioxidant responses, and metabolic functions. Additionally, specific microbial signatures associated with conditions like endometritis and respiratory disease suggest the potential for microbiota-based diagnostics and therapeutics. The identification of antibiotic-resistant strains of *Proteus mirabilis* and *Klebsiella pneumoniae* in donkey meat highlights food safety concerns requiring enhanced monitoring systems and standardized safety protocols. These findings provide a foundation for improved donkey healthcare management, including targeted disease surveillance, microbiota-based interventions, and protective measures for those working with donkeys or consuming donkey-derived products.

## 1. Introduction

Globally, the donkey population faces endangerment due to insufficient conservation efforts and limited utilization of donkey-derived products [1,2,3]. In contrast, China has recently directed substantial research attention toward donkeys, encompassing conservation initiatives, healthcare protocols, and product enhancement strategies [1,2,3,4,5,6]. This increased focus appears to be primarily driven by growing market demands for donkey-derived products, including ejiao (a traditional Chinese medicine produced from skin gelatin) [7,8,9], milk [10,11,12,13,14,15,16], and meat [17,18,19,20,21]. The production of high-quality donkey-derived commodities necessarily depends on optimal donkey healthcare as a fundamental prerequisite that warrants comprehensive consideration [3,4,5,6].

Recent studies have revealed significant health challenges facing donkey populations in China, particularly concerning infectious diseases and owner awareness [4,6]. Many health issues including reproductive disorders and viral, bacterial, and parasitic diseases are prevalent in various regions of Chinese donkey populations [22,23,24,25,26]. The intricate relationship between microbiota and host health has emerged as a critical area of research across species. In donkeys, these microbial communities play fundamental roles in maintaining physiological homeostasis, supporting digestive functions, and contributing to overall well-being [27,28,29].

Recent advancements in molecular techniques have enhanced our understanding of the diverse microbial ecosystems inhabiting various body sites in donkeys and their profound impact on health outcomes [27,28,29]. Donkeys, as single-stomach herbivores, possess complex microbial communities throughout their digestive tracts that are essential for intestinal homeostasis and nutritional processing [28,29]. Research has revealed significant variations in microbiota composition across different intestinal segments, with the hindgut displaying greater richness and diversity compared to the foregut [30,31,32]. This spatial distribution reflects specialized metabolic functions, with foregut microbiota primarily involved in carbohydrate metabolism and hindgut communities more active in amino acid metabolism [30,32].

Beyond the digestive system, distinct microbial profiles have been characterized across various body sites including the skin [33], oral cavity [34,35], reproductive tract of donkeys [36], as well as bodily secretions such as milk [37,38]. These site-specific communities contribute to local immune function, barrier protection, and physiological processes. Age-related and seasonal variations in microbiota composition have also been documented, demonstrating the dynamic nature of these microbial ecosystems throughout the donkey’s lifespan [39,40,41].

The health implications of donkey microbiota extend to critical areas including nutrition, immune function, and disease susceptibility [42,43,44]. Research has demonstrated how dietary interventions, environmental stressors, and physiological states like pregnancy can significantly alter microbial communities [45,46,47,48]. These alterations, in turn, correlate with changes in inflammatory markers, antioxidant responses, and metabolic functions, highlighting the microbiota’s central role in health regulation. Additionally, emerging research has identified specific microbial signatures associated with various health conditions in donkeys, from reproductive disorders to respiratory ailments [38,49]. This growing body of evidence points toward the future development of microbiota-based diagnostic and therapeutic approaches in donkey healthcare.

As donkey populations face numerous health challenges, including viral, bacterial, and parasitic diseases, understanding the protective and regulatory roles of commensal microbiota becomes increasingly important. The complex interplay between pathogenic agents and resident microbiota may offer new insights into disease prevention and management strategies. This review synthesizes current knowledge on donkey microbiota across body sites, explores their functional significance, and examines their relationships with health and disease, providing a foundation for future research and practical applications in donkey healthcare.

## 2. Literature Search Methodology

Data from articles published between 1 March 2017 and 1 March 2025 were selected for this review. Literature searches were conducted using the following keywords: “donkey health”, “parasitic diseases”, “viral diseases”, “bacterial diseases”, “microbiota”, and “China”. For this comprehensive review, we conducted a systematic search across multiple scholarly databases, including Web of Science, Google Scholar, PubMed, and Scopus, to identify and synthesize relevant literature. Only peer-reviewed articles indexed in the Science Citation Index (SCI) and published in English were included in this analysis. To maintain scholarly rigor, books, book chapters, conference proceedings, articles published in languages other than English and unpublished materials were excluded from the review.

## 3. Donkey Disease Surveillance Research in China

Various viral, bacterial, and parasitic diseases reported in the Chinese donkey population over the last seven years are summarized in Table 1. China’s strategic investment in donkey disease surveillance has revealed a complex epidemiological landscape threatening these economically valuable animals. The comprehensive data on donkey health issues, particularly from China, reveal a complex landscape of health challenges affecting both animal welfare and public health. These health issues span viral, bacterial, and parasitic diseases, each presenting distinct concerns that warrant careful consideration from both veterinary and public health perspectives. Notably, Deng et al. [6] identified a crucial gap in owners’ understanding of prevalent donkey diseases, emphasizing the urgent need for improved management and routine healthcare practices in northeastern China. This knowledge deficit becomes particularly concerning when considered alongside the emerging viral threats in the region.

In the realm of viral pathogens, Equine herpesviruses (EHV) stand out as significant concerns, with EHV-8 and EHV-1 causing a range of clinical manifestations including viral encephalitis, abortion, and respiratory distress [50,51,52,53]. Of particular significance is the widespread presence of Equine herpesviruses (EHV), which have emerged as major pathogens affecting donkey health and reproduction. A comprehensive surveillance study in Liaocheng, a primary donkey trading hub in Shandong Province, demonstrated an alarming EHV prevalence of 62.96% among large-scale farms, with heightened infection rates observed in donkeys aged 1–4 years during fall/winter seasons [23]. This finding gains additional importance when considered alongside the groundbreaking identification of Equine herpesvirus type 1 (EHV-1) in donkeys, specifically as a cause of abortions in China [51]. Further substantiating these concerns, researchers documented EHV-1 isolation from respiratory tract samples of donkey foals presenting with respiratory distress and high fever [50]. Equine herpesvirus type 8 (EHV-8), has emerged as a substantial threat to the global equine industry, causing considerable economic losses through its association with abortion, respiratory symptoms, and viral encephalitis [52,53]. In a noteworthy case study, researchers isolated EHV-8 from the brain of a deceased 2-year-old male donkey that exhibited severe neurological disorders, with subsequent confirmation through PCR and immunohistochemistry [53]. The significance of this pathogen is further emphasized by the high prevalence rate of 38.7% (457/1180) in donkey populations [54].

Recent experimental studies have advanced our understanding of EHV-8 pathogenesis. Investigation of EqHV-8 infection in C57BL/6J mice revealed significant clinical manifestations, including weight loss, dyspnea, and viremia, accompanied by elevated pro-inflammatory cytokine expression in brain and lung tissues [55]. These findings align with earlier research documenting increased levels of IL-6, IL-1β, and TNF-α in EqHV-8 infected mouse lung models [56]. Notably, Wang et al. [57] discovered that HO-1, an antioxidant defense enzyme, inhibits EqHV-8 replication through its metabolite biliverdin, operating via PKCβ/ERK1/ERK2 and NO/cGMP/PKG signaling pathways. Recent research has made notable progress in treatment approaches, with studies demonstrating promising results using compounds such as rutin, blebbistatin, hyperoside, and CoPP [58,59,60,61].

The viral disease landscape in donkeys extends beyond herpesviruses. Researchers have documented the presence of Hepatitis E virus (HEV) [62] and identified a novel astrovirus (DAstV-1) associated with severe diarrhea in donkey foals [63]. Of particular significance to public health is the identification of Hepatitis E virus genotypes 3 and 4 in donkeys, as documented by Rui et al. [62], which represents a considerable zoonotic risk, especially in areas where human-donkey contact is frequent.

Concurrent with viral threats, parasitic infections pose significant challenges, including *Giardia duodenalis* [64], piroplasmosis caused by *Theileria equi* and *Babesia caballi* [65], *Enterocytozoon bieneusi* [66], and various *Entamoeba* species [67]. Recent investigations have also identified three Cryptosporidium species alongside *Giardia duodenalis* and *Enterocytozoon* in Inner Mongolia [68]. Parasitic diseases documented in donkeys present perhaps the most extensive public health implications. Researchers have identified *Giardia duodenalis* and Cryptosporidium spp. across multiple regions including Xinjiang, Gansu province, and Inner Mongolia [25,68], with these parasites capable of causing significant diarrheal illness in both donkeys and humans. *Enterocytozoon bieneusi*, found throughout various provinces including Shandong, Jilin, and Liaoning [66], represents a significant opportunistic pathogen, particularly concerning for immunocompromised individuals. The isolation of *Toxoplasma gondii* from donkey serum, meat, and milk across multiple provinces [69,70] presents notable risks to public health, especially for vulnerable populations such as pregnant women and immunocompromised individuals.

The bacterial pathogen landscape presents equally significant challenges, with the discovery of antibiotic-resistant strains of *Proteus mirabilis* and *Klebsiella pneumoniae* in donkey meat from Beijing raising substantial food safety concerns [71]. The presence of *Proteus mirabilis* and *Klebsiella pneumoniae*, as reported by Liu et al. [71], further emphasizes the zoonotic potential of these bacterial infections and their importance to public health surveillance. In the bacterial disease spectrum, strangle associated with *Streptococcus equi subspecies equi* has been documented as a significant concern [72]. These findings have far-reaching implications for public health management and policy. The presence of antibiotic-resistant bacteria in donkey meat necessitates enhanced food safety measures, while the detection of *T*. *gondii* in donkey milk and meat products calls for rigorous monitoring. The identification of numerous zoonotic pathogens in donkeys necessitates comprehensive protective measures for individuals working closely with these animals, including testing and treating only affected individuals when proven-effective drugs are available and strict hygiene practices to address the multiple parasitic species with human transmission potential. An effective public health response requires enhanced surveillance systems integrating veterinary, environmental, and human health monitoring, standardized safety protocols for donkey handlers and product processors, rigorous quality control for donkey products entering human consumption channels, and education programs targeting workers and consumers. These findings warrant greater attention due to the expanding commercial importance of donkey-derived products globally, with future initiatives needing to address specific transmission pathways between donkeys and humans, differential disease severity across population groups, regional surveillance programs focusing on high-risk areas, and occupational safety regulations for the growing donkey industry. Implementing a One Health framework would strengthen the coordinated response to these zoonotic threats, ensuring continued safe utilization of donkeys in agriculture, food production, and traditional medicine contexts.

**Table 1:** Summary of donkey disease surveillance research in China.

Causative Agent	Clinical Manifestations/Findings	Treatment	Host/Model System	Reference
Bacterial pathogens				
*Salmonella abortus equi*	61 cases of abortion *S*. *abortus equi* was confirmed through serological and molecular testing	Minocycline	Donkey	[26,73]
Antibiotic resistance bacteria (*Proteus mirabilis* and *Klebsiella pneumoniae*) isolated from donkey meat	Potential public health concern		Donkey	[71]
*Coxiella burnetii*; *Salmonella*	Isolated *S*. *abortus equi* from 45 donkeys that experienced abortions *C*. *burnetii* isolated from 17 donkeys with abortion and confirmed through real-time PCR		Donkey and mouse	[74,75]
*Streptococcus equi Subspecies zooepidemicus*, *Escherichia coli* and *Acinetobacter* spp.	Serological, histopathalogical, and molecular diagnosis of endometritis induced bacteria		Donkey	[38,76,77,78,79]
*Streptococcus equi*	Strangles with fever and respiratory distress Isolated *S*. *equi* from strangles epidemic on donkey farms		Donkey	[80,81]
Viral pathogens				
EHV8	Viral encephalitis with neurological disorder		Mouse and Donkey	[53]
EHV8	Abortion and respiratory distress		Donkey	[23,54]
EHV8	Inflammation and respiratory distress		Mouse lung	[56]
EHV8	Abortion		Donkey	[52]
EHV1	Abortion and respiratory distress		Mouse and Donkey	[50,51]
EHV8	Significantly reduced inflammation and oxidative stress in mouse lung by activating AMPK and Nrf2/HO-1 signaling pathways	Cepharanthine	Mouse, NBL-6, and RK-13 cells.	[82]
EHV8	Inhibited virus infection	Blebbistatin	Mouse, rabbit kidney (RK-13), and Madin–Darby Bovine Kidney (MDBK) cells	[59]
EHV8	CoPP induced HO-1 inhibit EqHV-8 replication in susceptible cells through its metabolite biliverdin, which acts via PKCβ/ERK1/ERK2 and NO/cGMP/PKG signaling pathways	CoPP	Mouse, NBL-6, and RK-13 cells.	[57,61]
EqHV-8	Rutin prevented EqHV-8 induced infection and oxidative stress via Nrf2/HO-1 signaling pathway	Rutin	Mouse, MDBK, and RK-13 cells.	[58]
Hepatitis E virus genotypes 3 and 4	Potential public health concern		Donkey	[62]
Kirkoviruses	Intestinal inflammation and Diarrhea		Donkey	[83]
Astro-virus	Diarrhea		Donkey	[63]
Equine corona virus	Fever, anorexia, and diarrhea		Donkey	[84]
Rotavirus	Enteritis		Donkey	[85]
Equine influenza virus (H3N8 subtype)	High fever, cough, nasal discharge, enteritis, and abortion		Donkey	[86,87]
Porcine circovirus 3	Reproductive disorders including abortion		Donkey	[88]
Parasitic pathogens				
*Tetratrichomonas buttreyi* and *Pentatrichomonas hominis*	Causes diarrhea and has potential for zoonotic transmission		Donkey	[89]
*Giardia duodenalis* and *Cryptosporidium* spp.	Diarrhea and potential public health concern		Donkey	[25,90]
*Giardia duodenalis*	Diarrhea and potential public health concern		Donkey	[64]
*Entamoeba* sp. *RL9* and *Entamoeba equi*	Diarrhea and potential public health concern		Donkey	[67]
*Theileria equi*, *Babesia caballi*	Piroplasmosis (fever, anaemia, oedema, weight loss, icterus)		Donkey	[65,91]
*Theileria equi*, *Babesia caballi*	Piroplasmosis (fever, anaemia, oedema, weight loss, icterus)		Donkey	[92]
*Cryptosporidium* spp., *Giardia duodenalis* and *Enterocytozoon bieneusi*	Diarrhea and potential public health concern		Donkey	[68]
*Sarcocystis species* (*Sarcocystis bertrami*, *S. equicanis* and *S. fayeri*)	Muscle damage, myositis, encephalitis, diarrhea, and weight loss		Donkey	[93,94]
*Enterocytozoon bieneusi* and *Blastocysti*	Diarrhea and potential public health concern		Donkey	[95]
*Parascaris univalens* and *Parascaris equorum*	Hepatitis, pneumonitis, respiratory disorders, intestinal obstruction, and even mortality if their hosts are untreated		Donkey	[96]
*Enterocytozoon bieneusi* and *Giardia duodenalis*	Diarrhea and potential public health concern		Donkey	[97]
*Enterocytozoon bieneusi*	Diarrhea and potential public health concern		Donkey	[98]
*Cryptosporidium*	Diarrhea and potential public health concern		Donkey	[99,100]
*Toxoplasma gondii* isolated from serum, meat and milk of donkey	Swollen lymph nodes, headaches, fever, fatigue, abortion, and muscle aches and pains		Donkey	[69,101,102,103]
*Enterocytozoon bieneusi*	Diarrhea and potential public health concern		Donkey	[66]
*Neospora* spp. (*N. caninum*)	Miscarriages, myositis, and pneumonia		Donkey	[104]
*Habronema muscae* and *H. majus*	Diarrhea and intestinal ulceration		Donkey	[105]
High concentrate feeds, Age pasture time and water source	Infundibular caries		Donkey	[106]

## 4. The Intestinal Microbiota of Donkeys: Distribution, Function, and Physiological Significance

Donkeys, as single-stomach herbivores, possess a complex and diverse microbial community in their digestive tracts. The intestinal bacterial community plays a crucial role in maintaining intestinal homeostasis, as well as the host’s overall nutrition and health [28,31,39]. Recent investigations have revealed significant variations in donkey intestinal microbiota across different intestinal sites, physiological states, and geographical regions, highlighting the complex nature of the equine gut microbiome and its adaptations to various anatomical locations, physiological changes, and regional environmental conditions [30,31,32,33,34,35,41].

### 4.1. Microbiota Composition of Different Intestinal Segments

**Figure 1:**
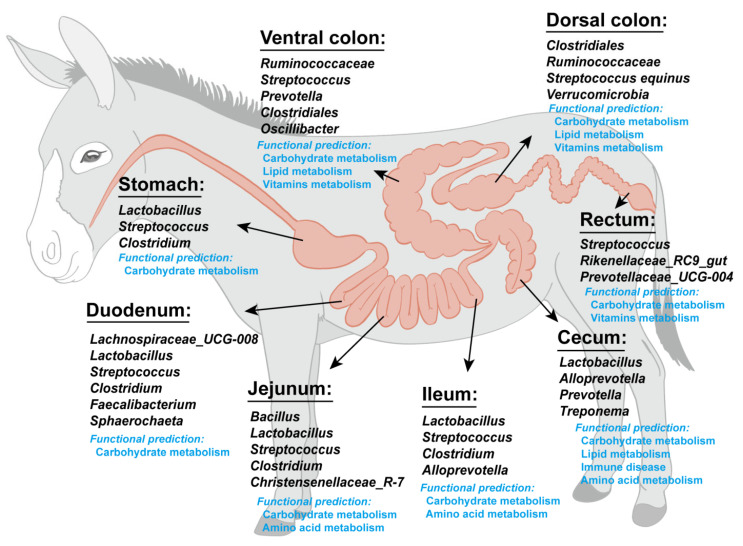
Donkey intestinal microbiota composition and their functional prediction in different intestinal segments.

The distinct microbial communities colonizing various segments of the donkey intestinal tract exhibit significant taxonomic and functional diversity, reflecting specialized adaptations to the unique physiological conditions of each digestive compartment (Figure 1). Consistently, Wang et al. have documented distinct variations in microbiota composition across various parts of the intestine in donkeys [31]. Correspondingly, a comprehensive study explored the microbiota of the donkey hindgut and identified specific bacterial genera in the cecum (such as *Prevotella*, *Desulfovibrio*, *Alistipes*, and *Treponema_D*) and nine metagenome-assembled genomes (MAGs) in the dorsal colon (such as *Limimorpha*, *Saccharofermentans*, and *Lactobacillus*) that were associated with complex carbohydrate degradation and hindgut metabolism [27]. The microbiota of healthy donkeys exhibits higher richness and diversity in the hindgut compared to the foregut, with Firmicutes dominating the foregut and both *Firmicutes* and *Bacteroides* demonstrating abundance in the hindgut [32]. At the genus level, *Lactobacillus* predominates in the foregut, while *Streptococcus* prevails in the hindgut. Functional analysis has revealed that foregut microbiota is more involved in carbohydrate metabolism, whereas hindgut microbiota demonstrates greater activity in amino acid metabolism [32]. Furthermore, a study on donkey hindgut microbiota reveals significant insights into the role of microbiota in immune response. A notable difference in dominant bacterial communities across different sections of the donkey hindgut were revealed, with species like *Prevotella* and *Treponema* dominating the cecum, while *Clostridiales_bacterium* and *Streptococcus_equinus* were more prevalent in the dorsal colon. These microbial variations corresponded with differences in short-chain fatty acid concentrations, where except for propionate, levels of acetate, isobutyrate, valerate, and isovalerate were lower in the cecum compared to the dorsal colon [29]. Importantly, they identified differentially expressed genes related to immune function between these gut regions, including mucin genes (*MUC3B*, mucin-2-like), interleukin-related genes (*IL17RC*, *IL1R2*, *IL33*), complement system components (*C1QA*), and matrix metalloproteinases (*MMP9*). The Peroxisome Proliferator-Activated Receptor (PPAR) pathway was particularly enriched in the cecum, suggesting it plays a crucial role in mediating interactions between gut microbiota and immune function. This complex relationship between microbial communities and host gene expression indicates that regional microbiota influences the mucus layer differently, affect inflammatory and immune signaling pathways, and maintain gut homeostasis through mechanisms involving the PPAR pathway [29].

Research has demonstrated that bacteria in the adherent (Ad) fraction of the donkey hindgut exhibited higher diversity than those in the liquid (Lq) fraction. *Bacteroidota*, *Spirochaetota*, *Fibrobacterota*, and *Patescibacteria* showed greater abundance in the Ad fraction, indicating their significant role in plant fiber degradation [107]. Additionally, they reported that *Lactobacillus* was more abundant in the Lq fraction, suggesting enhanced hydrolysis of fermentable carbohydrates [107]. Consistently, it has been documented that the hindgut of donkeys has significantly higher microbiota diversity and richness than the foregut, with no sex-related differences [108]. Furthermore, the donkey hindgut represents a particularly microbial-rich environment, with the caecum and colon playing pivotal roles in dietary fiber degradation through fibrolytic enzymes. Spectrophotometric measurements have revealed higher fibrolytic enzyme activity in the dorsal colon compared to the caecum [109]. Fungal community analysis identified *Ascomycota*, *Basidiomycota*, and *Neocallimastigomycota* as the predominant phyla, with genera such as *Aspergillus* and *Fusarium* being dominant [109]. Notably, *Neocallimastigomycota* and enzymes involved in plant cell wall breakdown were more abundant in the colon, suggesting its central role in fiber degradation processes [109].

The intestinal microbiota of donkeys demonstrated a dominance of *Firmicutes* and *Bacteroidetes* across all intestinal segments, with starch-degrading bacteria like Lactobacillus enriched in the small intestine and fiber-degrading bacteria like *Akkermansia* in the large intestine [110]. Metabolic functions for lipid metabolism and membrane transport were more prominent in the small intestine, while energy and amino acid metabolism were enriched in the large intestine. Differences in microbial composition were more pronounced in the digesta-associated microbiota, highlighting the distinct functional roles of small and large intestines [110].

### 4.2. Composition of the Gut Microbiota at Different Physiological Stages

Developmental changes in microbiota have also been documented, particularly in the oral cavity. A longitudinal study revealed that the oral microbiota diversity in donkey foals significantly increased after weaning, with higher Simpson index values observed in postweaning foals [34]. At the phylum level, *Firmicutes* and *Myxococcota* demonstrated greater abundance postweaning, while genera such as *Gemella*, *Lactobacillus*, and unclassified *Lactobacillales* showed notable increases [34]. Functional analysis further indicated that carbohydrate metabolic pathways were significantly enriched in the oral microbiome after weaning, reflecting dietary transitions [34]. It has been consistently shown that the gut microbiota of young donkeys exhibits lower diversity and richness compared to adults, with significant individual variation observed at 1 month of age [40]. During the aging process, the abundance of *Bacteroides*, *Lactobacillus*, and *Odoribacter* decreases, while *Streptococcus* sees a notable increase. Functional predictions have indicated age-related differences in microbial pathways, such as those involving Terpenoids and Polyketides, highlighting the distinct microbiota composition and functional stability that characterizes foals versus adult donkeys [40].

Reproductive status also significantly impacts microbiota composition in donkeys. During pregnancy, the intestinal microbiota of donkeys undergoes substantial changes, influencing growth, metabolism, immunity, and reproductive functions. Pregnant donkeys exhibited higher overall microbial richness, with the lowest diversity observed during early-stage pregnancy (EP) [47]. Specific families such as *Clostridiaceae* and *Streptococcaceae* were more abundant in EP, correlating with elevated inflammatory markers and altered serum biochemical parameters. These findings provide valuable new insights into the complex relationship between gut microbiota composition and reproductive health in donkeys [47]. It was further documented that the diversity of fecal bacteria in donkeys significantly increased throughout pregnancy. Phyla such as *Spirochaetota* and *Fibrobacterota*, and genera like *Treponema* and *Streptococcus*, showed significant changes in abundance during gestation [111]. Additionally, there were notable correlations between shifts in the gut microbiota and changes in plasma metabolites, supporting fetal development and maternal health [111]. In the present study, the microbiota of the different physiological stages was measured by targeted 16S rRNA gene (V3–V4 region) sequencing using the Illumina MiSeq. However, 16S rRNA sequencing has some limitations; for instance, it is difficult for the process to precisely distinguish differences at the species or strains, and it is unable to detect fungi, viruses, and protozoa, or to reveal specific functional genes or metabolic pathways of microorganisms [112]. The lack of species and functional information can be further compensated for with the use of metagenomic sequencing (functional annotation) and metabolomics profiling (metabolite validation).

### 4.3. Composition of the Gut Microbiota in Different Geographical Regions

Qinghai donkeys from the Tibetan Plateau exhibited higher microbial diversity than Dezhou donkeys, with *Bacteroidales* identified as major contributors to pathways involved in signal transduction and carbohydrate metabolism. This finding highlights their crucial role in the adaptation of Qinghai donkeys to extreme high-altitude environments [108]. Consistent with these observations, a comparative study revealed that Tibetan wild asses demonstrated significantly higher dry matter digestion capabilities than domestic donkeys and exhibited distinct gut microbiota composition on the Qinghai–Tibet plateau [113]. At the phylum level, *Bacteroidetes* and *Firmicutes* were more abundant in wild asses, with higher levels of genera like *Ruminococcaceae_NK4A214* and *Akkermansia*. Furthermore, wild asses possessed richer metabolic pathways related to amino acid, carbohydrate, and energy metabolism, suggesting an adaptive gut microbiome specifically suited for high-altitude environments [113].

Beyond the gastrointestinal tract, the microbiota of donkey milk displays significant temporal and geographical variations. Research has shown that donkey milk microbiota composition varies across lactation stages, with *Proteobacteria* and *Firmicutes* emerging as the dominant phyla [36]. The genera *Ralstonia*, *Pseudomonas*, and *Acinetobacter* were most abundant, while *Streptococcus* demonstrated increased presence in mature milk. Notably, pathogens such as *Escherichia-Shigella* and *Staphylococcus*, along with thermoduric bacteria, were also detected, highlighting both beneficial and potentially harmful microbial components [36]. Geographical influences on milk microbiota have also been documented. The bacterial communities in donkey milk from two distinct regions in China (Xinjiang and Shandong) were dominated by *Acinetobacter*, *Proteobacteria*, *Firmicutes*, and *Bacteroidetes*, but with significantly differing abundances between the groups [37]. Genera such as *Macrococcus* and *Acinetobacter* demonstrated higher abundance in Xinjiang milk, while *Streptococcus*, *Pseudoclavibacter*, and *Pseudomonas* were prevalent in Shandong samples. Alpha diversity analysis revealed significant differences in richness between the regions, highlighting potential microbial risks and opportunities for beneficial bacteria in donkey milk [37].

### 4.4. Influence of Diets and Environmental Stressors on the Composition of the Gut Microbiota

Nutritional interventions significantly impact the donkey microbiome and associated physiological parameters. Medium level energy supplementation (10.49 MJ/kg) for jennet donkeys during late gestation significantly enhances the rectal microbiota, with specific taxa such as *norank_f_norank_o_Coriobacteriales*, *norank_f_norank_o_Mollicutes_RF39*, *Candidatus_Saccharimonas*, and *Fibrobacter* showing positive associations with antioxidant enzyme activities including catalase (CAT), total superoxide dismutase (T-SOD), glutathione peroxidase (GSH-Px), and reduced inflammatory cytokines [43]. Further investigations revealed that the rectal microbiota was positively associated with average daily gain, reduced inflammatory cytokines, and enhanced antioxidant responses [43]. Consistent with these findings, another study demonstrated that methionine supplementation regulated specific microbiota components, including *Ruminococcus*, *Peptococcus*, *Anaeroplasma*, and *Methanocorpusculum*, which were positively correlated with total antioxidant capacity (T-AOC) and CAT activity. Additionally, *Peptococcus* showed a significant negative correlation with malondialdehyde (MDA) levels, a marker of oxidative stress [42]. Different feeding strategies have also shown significant effects on donkey microbiota composition. Fiber-to-concentrate (FC), concentrate-to-fiber (CF), and total mixed ration (TMR) feeding approaches significantly improved the abundance of beneficial bacteria such as *Prevotella*, *Bacteroides*, and *Fibrobacter* in the cecum of donkeys [45]. Similarly, multienzyme supplementation enhanced the levels of *Firmicutes*, *Oscillospiraceae*, *Lachnospiraceae*, *Christensenellaceae*, *Christensenellaceae_R-7_group*, and *Streptococcus* in feces, while simultaneously decreasing the abundance of *Proteobacteria* [46].

High-energy (HE) diets significantly altered the microbiome profile in donkeys, decreasing the *Firmicutes*-to-*Bacteroidetes* ratio and increasing the abundance of *Prevotellaceae*, while reducing the richness of *Ruminococcaceae* [114]. These dietary interventions also upregulated metabolic pathways related to aspartate metabolism and the urea cycle. Importantly, these microbial and metabolic changes demonstrated positive correlations with improved growth performance, suggesting that HE diets could effectively enhance feed efficiency and growth in donkeys [114]. In contrast, low-energy diets in meat donkeys reduced abundance of *Firmicutes* and *Actinobacteria* while increasing Bacteroidetes in the cecal microbiome, alongside higher levels of genera *Ruminococcaceae-UCG-004*, *Acinetobacter*, and *Rikenellaceae_RC9*_gut_group [115]. Furthermore, they revealed that the cecal metabolome showed upregulation of formyl-5-hydroxykynurenamine, chorismate, 3-sulfinoalanine, and 3-isopropylmalate, with downregulation of brassinolide, primarily affecting energy metabolism and oxidative stress pathways. These microbial and metabolic alterations in the cecum appear to be key mechanisms through which low-energy diets create negative energy balance, induce oxidative stress, and ultimately reduce growth performance in meat donkeys [115].

In line with these observations, a study demonstrated that yeast polysaccharide (YPS) supplementation (10 g/(jenny and their foal **•** d)) had significant positive effects on the gut microbiome of both Dezhou donkey foals and jennies, which correlated with enhanced immune function [116]. In foals, YPS promoted the growth of beneficial bacteria including *Lactobacillus* and *Prevotella*, which are known for their probiotic properties and ability to improve feed digestion. Similarly, in jennies, YPS increased the abundance of *Terriporobacter* and *Cellulosilyticum*, bacteria that contribute to enhanced feed utilization. These microbiota changes were associated with improved immune parameters, specifically elevated immunoglobulin A (IgA) and immunoglobulin G (IgG) levels in the jennies and increased complement component C4 concentrations in foals [116]. The relationship between gut microbiota and immunity was further supported by alterations in the plasma metabolome, particularly in lipids and lipid-like molecules. The study detected increased concentrations of specific metabolites such as 13,14-Dihydro PGF2a, 2-Isopropylmalic acid, and taurocholic acid, which may serve as mediators between the gut microbiota changes and improved immune function [116]. Similarly, total mixed ration feeding (mixture of roughage, concentrate, and water in equal proportions) significantly enhanced growth and digestibility in weaned foals by improving their fecal microbiota composition, particularly taxa such as *Treponema*, *Rikenellaceae-RC9*-gut-group, *Unidentified-F082*, and *Bacteroidales-RF16*-group [44].

Environmental stressors significantly impact donkey microbiota. Transportation-induced stress led to substantial decreases in bacterial richness in donkey fecal microbiota, with notable reductions in the abundance of *Atopostipes*, *Eubacterium*, *Streptococcus*, and *Coriobacteriaceae* post-transportation [117]. These microbiota changes, alongside increased stress markers such as cortisol, adrenocorticotropic hormone (ACTH), and heat shock protein 90 (HSP90), highlight the potential adverse impact of transportation on intestinal health in donkeys [117]. Consequently, a study reported that *Artemisia ordosica* crude polysaccharides (AOCP) supplementation positively altered the rectal microbiome, increasing diversity and reshaping the microbial community structure [118]. Notably, AOCP promoted the colonization of beneficial bacteria including *Lactobacillus*, *Unclassified_f_Prevotellacea*, *Ruminococcus*, and *Fibrobacter* genera, while simultaneously reducing pathogenic bacteria such as *Clostridium_sensu_stricto_1.* These microbiota changes correlated with enhanced immune parameters, specifically increased serum concentrations of immunoglobulins (IgA, IgG, and IgM), which are critical components of the humoral immune response [118]. The improved microbial profile also aligned with enhanced antioxidant status, as evidenced by increased activities of superoxide dismutase, catalase, and total antioxidant capacity, along with decreased concentrations of inflammatory markers including tumor necrosis factor-α, nitric oxide, reactive oxygen species, and malondialdehyde. The connection between microbiota and immune function was further supported by metabolomic analysis, which revealed AOCP-induced alterations in key metabolites involved in immunomodulatory pathways, including PPAR signaling, prolactin signaling, glycerophospholipid metabolism, and tyrosine metabolism. Additionally, AOCP supplementation increased rectal volatile fatty acid concentrations (propionate, butyrate, isovalerate, and total VFAs), which can serve as important energy sources for intestinal epithelial cells and modulate immune responses [118]. Consistent with these findings, another study reported that transportation significantly altered the nasal microbiota of donkeys, with increased abundance of *Proteobacteria* and decreased *Firmicutes* [48]. Principal coordinate analysis (PCoA) revealed structural changes in the microbiota following transportation, which correlated with elevated stress markers including cortisol and ACTH. These findings suggest that transportation-induced stress can substantially impact nasal microbiota composition and diversity, potentially influencing respiratory health outcomes [48].

### 4.5. Microbiota Composition and Donkey Diseases

The microbial landscape also plays a crucial role in reproductive health and pathogenesis in donkeys. The endometrial and vaginal microbiomes are particularly important in the context of donkey endometritis, a major cause of infertility. A comparative study of microbiomes from healthy jennies versus those with endometritis revealed significant microbial differences, especially in the abundance of *Ruminococcaceae* and *Lachnospiraceae* [38]. Furthermore, these findings suggest that microbiome imbalances, particularly those linked to anaerobic bacteria and *Sphingomonadaceae*, may contribute significantly to the pathogenesis of endometritis in donkeys [38]. In the context of respiratory pathogens, the nasopharyngeal microbiome of *Streptococcus equi* carrier donkeys showed a higher relative abundance of *Proteobacteria* and a lower abundance of *Firmicutes* and *Actinobacteria* compared to healthy counterparts [49]. Notably, *Nicoletella*, a genus newly detected in donkeys, dominated the microbiome in carriers and might suppress other normal upper respiratory tract bacterial community such as *Streptococcus* spp. and *Staphylococcus* spp. These microbial alterations indicate dysbiosis, which could potentially predispose carrier donkeys to additional respiratory diseases [49]. The summary of the factors affecting donkey microbiome and its association with metabolism and health is provided in Table 2.

## 5. Conclusions

Based on existing data we concluded that donkey populations in China face significant health challenges from viral, bacterial, and parasitic diseases. Equine herpesviruses (particularly EHV-8 and EHV-1) represent major threats, causing encephalitis, abortion, and respiratory distress. Parasitic infections like Giardia, Cryptosporidium, and Toxoplasma pose substantial zoonotic risks. Microbiota research in donkeys reveals critical relationships between gut bacteria and health, nutrition, immune function, reproductive success, environmental adaptation, and disease resistance, offering promising therapeutic potential for veterinary applications. Based on the comprehensive review, significant research gaps remain in understanding the interplay between microbiota dysbiosis and disease pathogenesis in donkeys. Future studies should focus on developing microbiome-based diagnostic tools and therapeutic interventions targeting the host-microbiome interface. Additionally, longitudinal studies examining how environmental factors, management practices, and geographical location influence donkey microbiome development would provide valuable insights for improving health outcomes and productivity in these economically important animals. Altogether, these findings underscore the urgent need for integrated disease surveillance, improved owner education, standardized healthcare protocols, and a One Health approach connecting veterinary and public health sectors to protect both donkey welfare and human health, especially as donkey-derived products gain commercial importance.

## Figures and Tables

**Table 2:** Effects of various treatments and factors on donkey gut microbiota composition and associated biological effects.

Treatment/Factors	Gut Microbiota	Biological Effect	Reference
Sex and intestinal segments	Male Duodenum and jejunum: *Streptococcus* and *Erysipelotrichaceae_UCG-002* Ileum: *Sarcina* and *Streptococcus* Female: Duodenum and jejunum: *Clostridium_sensu_stricto_1*, *Acinetobacter*, and *NK4A214* Ileum: Amnipila, Terrisporobacter, and Luteimonas	Health and sex-wise microbiota information	[30]
Intestinal segments	Duodenum: *Lachnospiraceae_UCG-008* and *Sphaerochaeta* jejunum: *Christensenellaceae_R-7_group* and *Bacillus* ileum: *NK4A214_group* and *Alloprevotella* UCG-005 Cecum; *Lactobacillus* Colon: *Clostridium_sensu_stricto_1* and *Chlamydia* Feces: *Rikenellaceae_RC9_gut_group* and *Prevotellaceae_UCG-004*	Body health, metabolism, and development of microbial additives.	[31]
Weaning	*Verrucomicrobiales*, *Clostridia*, *Oscillospiraceae*, *Akkermansia*, *Rikenellaceae*, *Clostridia Oscillospiraceae*, *Campilobacterota, Lachnoclostridium*, and *Roseburia.*	Metabolism and health	[39]
Methionine	*Ruminococcus*, *Peptococcus*, *Anaeroplasma*, and *Methanocorpusculum*	Antioxidant response and health	[42]
*Artemisia ordosica* crude polysaccharides	Improved colonization of beneficial bacteria, including *Lactobacillus*, *Unclassified_f_Prevotellacea*, *Ruminococcus*, and *Fibrobacter genera.* Decreased pathogenic bacterial colonization of the *Clostridium_sensu_stricto_1 bacterial* genus	Improved antioxidant response, lactational performance, and health	[118]
*Corn Silage*	Enhanced Bacteroidetes (Genera *Prevotellaceae_UCG-003*, *Alloprevotella* and *Prevotella_1*) and Firmicutes phyla (Genera *Ruminococcaceae_NK4A214_group*, *Ruminococcaceae_UCG-010*, *Lachnospiraceae*, and *Ruminococcaceae_UCG-002*)	Metabolism and intestinal health	[119]
Fibrolytic enzyme	Predominant fungi at phylum level were *Ascomycota*, *Basidiomycota*, and *Neocallimastigomycota.* *Aspergillus*, *Wallemia*, *Phanerochaete*, *Fusarium*, and *Penicillium* were detected as the dominant genera	Plant cell wall breakdown and digestion	[109]

## Data Availability

No new data were created or analyzed in this study.
